# The Leucine-Rich Repeat Kinase 2 Variant LRRK2^G2019S^ Up-Regulates L-Type (CaV1.3) Calcium Channel via the Ca_V_β_3_ Subunit: Possible Role in the Pathogenesis of Parkinson’s Disease

**DOI:** 10.3390/ijms26073229

**Published:** 2025-03-31

**Authors:** Alejandro Sandoval, Alejandra Corzo-López, Paz Duran, Diana Tovar-Soto, Bryan Vargas-Caballero, Valeria Galicia-Saldaña, Ricardo González-Ramírez, Ricardo Felix

**Affiliations:** 1 School of Medicine FES Iztacala, National Autonomous University of Mexico (UNAM), Tlalnepantla 54090, Mexico; maria.corzo@cinvestav.mx (A.C.-L.); dianaj.tovars@gmail.com (D.T.-S.); valeria.sgalicia@gmail.com (V.G.-S.); 2 Department of Molecular Pathobiology, College of Dentistry, New York University, New York, NY 10010, USA; duran.paz9@gmail.com; 3 Department of Cell Biology, Centre for Research and Advanced Studies (Cinvestav), Mexico City 077360, Mexico; bryan.vargas@cinevstav.mx; 4 Department of Molecular Biology and Histocaompatibility, “Dr. Manuel Gea González” General Hospital, Mexico City 14080, Mexico; grricardo_gr@yahoo.com.mx; 5 Center for Research on Aging, Cinvestav, Mexico City 14330, Mexico

**Keywords:** calcium channels, Cav1.3, LRRK2, Parkinson’s disease

## Abstract

Voltage-gated Ca^2+^ (Ca_V_) channels are transmembrane proteins comprising the pore-forming subunit Ca_V_α_1_ and the ancillary proteins Ca_V_α_2_δ and Ca_V_β. They are expressed in various tissues, including the nervous system, where they regulate Ca^2+^ entry in response to membrane potential changes. The increase in intracellular Ca^2+^ allows for regulating cell excitability and releasing neurotransmitters, among other cellular events. Leucine-rich repeat kinase 2 (LRRK2) is a serine–threonine kinase involved in vesicular mobilization. Previously, it has been shown that LRRK2 regulates neurotransmission by phosphorylating the Ca_V_β auxiliary subunit of the Ca_V_2.1 (P/Q-type) presynaptic channels. However, it is unknown whether the kinase can regulate the activity of other Ca_V_ channel subtypes, such as Ca_V_1.3 (L-type), which play a significant role in the excitability of dopaminergic neurons in the substantia nigra pars compacta (SNc) and whose dysregulation contributes to neurodegeneration in Parkinson’s disease (PD). Here, we found potential phosphorylation sites for LRRK2 in Ca_V_β_3_ and examined how these molecules interact. We used immunoprecipitation and electrophysiology in HEK-293 cells expressing recombinant Ca_V_1.3 channels, both with and without wild-type LRRK2 or its LRRK2^G2019S^ mutation, which plays a role in familial PD through a possible gain-of-toxic-function mechanism. Our results show that LRRK2^G2019S^ significantly increases current density through Ca_V_1.3 channels, and this effect depends on the presence of Ca_V_β_3_. Site-directed mutagenesis revealed that phosphorylation at S152 in the sequence of Ca_V_β_3_ is necessary and sufficient to explain the abnormal regulation of the channels mediated by LRRK2^G2019S^. These data provide new insights into the molecular regulation that mutant LRRK2 may exert on L-type Ca_V_1.3 channels, which determine pacemaker activity in dopaminergic neurons of the SNc and may, therefore, play a relevant role in the molecular pathophysiology of PD.

## 1. Introduction

Voltage-dependent calcium (Ca_V_) channels are transmembrane proteins that facilitate calcium entry into cells in response to depolarizations in the membrane. Channel proteins are expressed in diverse cell types, including neurons, endocrine cells, and muscle tissue, where they transduce electrical signals from the cell surface into chemical messages that regulate various functions such as neurotransmission, hormonal secretion, gene transcription, and muscle contraction [1,2]. Ca_V_ channels are composed of an ion-conducting subunit called Ca_V_α1, along with at least two auxiliary subunits, Ca_V_β and Ca_V_α_2_δ.

Ca_V_ channels are classified into low voltage-activated (LVA) and high voltage-activated (HVA) channels based on their activation thresholds. HVA channels are further divided into four types: L, N, P/Q, and R. The L-type channels consist of four varieties: Ca_V_1.1 to Ca_V_1.4 [2,3]. L-type channels of the Ca_V_1.3 class are widely expressed in the brain tissues, inner ear receptors, the sinoatrial node, and endocrine cells. In the brain, they contribute to developing fear memory, long-term potentiation, and emotional behavior. In the sinoatrial node, Ca_V_1.3 channels determine the functional properties of pacemaker cells, while in the inner ear receptors, they are responsible for the release of neurotransmitters [4]. Ca_V_1.3 channels also contribute to the secretion processes in β pancreatic cells and the peacemaking activity of chromaffin cells [5,6]. The Ca_V_1.3α_1_^-/-^ mice present deafness and cardiac arrhythmias [7], while in humans, mutations in the Ca_V_1.3α_1_ subunit have been associated with the syndrome of sinoatrial node dysfunction, deafness, and hyperaldosteronism. Interestingly, Ca_V_1.3 channels may play a role in the pathophysiology of neuropsychiatric disorders and the neurodegenerative process of Parkinson’s disease (PD) [3,8].

Phosphorylation is one of the key mechanisms for regulating ion channel activity. Ca_V_ channels are the targets of various kinases, such as PKA, PKC, PKG, and CamKII, among others [1,5]. Previously, it was found that P/Q-type calcium channels, expressed in HEK-293 cells, can be regulated by the leucine-rich repeat kinase 2 (LRRK2) [9]. LRRK2 is a large protein of 2526 aa that, in addition to having a kinase domain, has GTPase capacity through which it autophosphorylates and forms dimers [10]. It also contains multiple domains for interaction with other proteins and is expressed in several organs and tissues, including the central nervous system (CNS) [11]. While the primary function of LRRK2 remains unclear, evidence suggests a role in dendrite development, neuronal maturation, synaptic transmission, and autophagy. Likewise, LRRK2 interacts with molecules related to vesicle mobilization [12]. The kinase also influences the expression of Na^+^/Ca^2+^ exchangers in dendritic regions, contributing to the buffering of free Ca^2+^ [13]. Additionally, LRRK2 modifies the expression of Ca_V_ channels, increasing current density and causing a hyperpolarization of the voltage dependence of channel activation, increasing the number of channels available to be opened from more negative voltages [9].

These effects are specific to LRRK2, as LRRK1 does not show them. They are more pronounced with the LRRK2 activity-enhanced mutant LRRK2^G2019S^ and are reduced by the inhibitors. These results support the idea that PD originates from a disorder of Ca^2+^ homeostasis. In this case, an increase in Ca^2+^ entry may occur, which cannot be appropriately counteracted by the Ca^2+^ buffering mechanisms. This is followed by an increase in oxidative stress, mitochondrial alterations, proteasomal dysfunction, and excitotoxicity. These conditions contribute to premature aging and, ultimately, the death of dopaminergic neurons in the substantia nigra pars compacta (SNc). However, it is unknown whether LRRK2 can regulate the activity of other Ca_V_ channels, particularly Ca_V_1.3, which are highly expressed in these neurons and are considered the most involved in the Ca^2+^-dependent pathophysiology of PD. A precise understanding of the functioning of Ca_V_1.3 channels and their regulation by different signaling pathways clarifies their contribution to the excitability of dopaminergic neurons in the SNc and possible role in the molecular pathophysiology of PD.

## 2. Results

### 2.1. The Subunits of L-Type Ca_V_1.3α Channels May Contain Motifs for Regulation by LRRK2

Initial bioinformatic analysis identified various consensus phosphorylation sites [14] of the serine/threonine protein kinase LRRK2 in the sequences of both the Ca_V_1.3α_1_ and Ca_V_β_3_ subunits. This was possible using a free access database at the URL https://gps.biocuckoo.cn/. Specifically, we were able to identify one phosphorylation site (Ser793) in the ion-conducting subunit (Ca_V_1.3α_1_) and four in the Ca_V_β_3_ auxiliary subunit (Ser152, Ser245, Thr283, and Thr316).

### 2.2. Expression of LRRK2 and Its Increased Activity Mutant LRRK2^G2019S^

**Figure 1:**
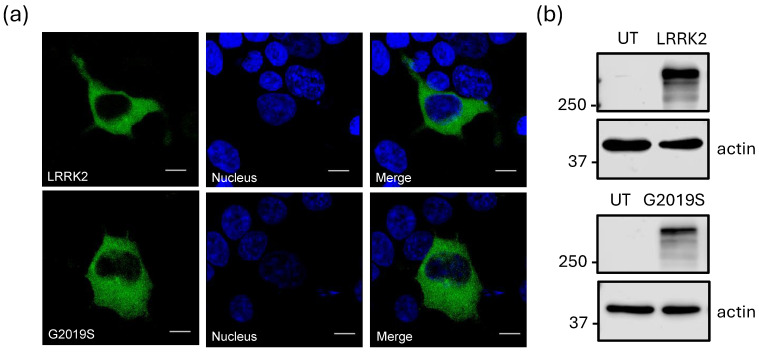
Heterologous expression of LRRK2 and its increased activity mutant LRRK2G2019S. (**a**) Confocal images of HEK-293 cells expressing the two kinase variants fused to GFP. The nuclei are stained with DAPI (blue). The upper panels show overexpression of the LRRK2^WT^ protein, while the lower panels illustrate the expression of the LRRK2^G2019S^ variant. The far-right panels display the merged images for both constructs. The data shown are representative of three separate experiments. Scale bar = 10 μm. (**b**) Protein expression levels were evaluated in extracts from untransfected (UT) HEK-293 cells or cells co-expressing the variants of the LRRK2 kinase. Protein extracts were subjected to Western blot analysis using the antibodies listed in Supplementary Table S1. The molecular weight markers are shown on the left. Actin served as a loading control.

To assess the expression of the two variants of the LRRK2 kinase in the heterologous system used in this study, we employed two distinct detection methods using GFP-tagged constructs of LRRK2^WT^ and LRRK2^G2019S^. First, confocal microscopy revealed a signal consistent with a diffuse cytosolic distribution of the proteins of interest using both constructs (Figure 1a). Unlike what was observed in previous reports indicating abnormal accumulation of LRRK2^G2019S^ in the nucleus and Golgi apparatus [15,16], our results suggested that the presence of the mutation does not affect its subcellular localization. Similarly, Western blot analyses demonstrated comparable levels of LRRK2 expression between the wild-type and mutant proteins (Figure 1b). These findings indicate that, at least under this experimental context, the presence of the mutation does not have an impact on the overall expression or distribution of LRRK2 within HEK-293 cells.

### 2.3. LRRK2 Interacts with Ca_V_β_3_ Subunit in HEK-293 Cells

**Figure 2:**
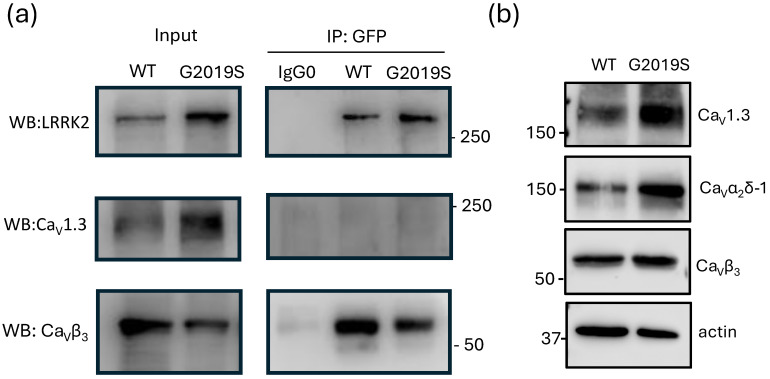
LRRK2 interacts with the Ca_V_1.3 (L-type) channel complex. (**a**) Proteins from HEK-293 cells transiently transfected with the two kinase variants (WT and G2019S), along with the Ca_V_1.3α_1_, Ca_V_β_3_, or Ca_V_α_2_δ-1 channel subunits, were subjected to immunoprecipitation (IP) assays using anti-GFP antibodies. The anti-EspC antibody, which is directed against a serine protease autotransporter protein secreted by enteropathogenic E. coli, was utilized as an irrelevant control antibody (IgG 0). The immunoprecipitated proteins were subsequently analyzed using Western blot with specific antibodies as indicated. The auxiliary subunit Ca_V_β_3_ was IP using the anti-GFF antibody (bottom panel). In contrast, the pore-forming subunit Ca_V_1.3α_1_ did not show interaction with the kinase (middle panel). (**b**) Western blot analysis was conducted to investigate the actions of the two LRRK2 variants on the expression levels of proteins that form the L-type Ca^2+^ channel complex, Ca_V_1.3. Actin served as a loading control. The data shown represent findings from three independent experiments.

Previous work by Bedford et al. (2016) showed that LRRK2 can directly associate with the Ca_V_β_3_ subunit, linking it functionally to the voltage-gated Ca^2+^ channels of the Ca_V_2.1 class (P/Q-type) [9]. To determine if this mechanism also applies to L-type channels of the Ca_V_1.3 class, we conducted experiments using specific antibodies against GFP, Ca_V_1.3α_1_, and Ca_V_β_3_. We aimed to determine whether LRRK2 could coimmunoprecipitate with the L-type channel complex from lysates of cells heterologously expressing the proteins of interest. We used anti-GFP antibodies in this experiment because, as mentioned in Methods, both LRRK2 variants were fused to GFP. As illustrated in Figure 2a, immunoprecipitation using anti-GFP antibodies revealed a band that exceeds the 250 kDa size marker, corresponding to an expected molecular weight of 313 kDa. This size comprises 286 kDa from the kinase and 27 kDa from the GFP tag. The results were consistent for LRRK2^WT^ and the mutant LRRK2^G2019S^ protein, validating the selectivity of the antibodies used in our analysis. Interestingly, immunoprecipitation assays using anti-GFP antibodies did not yield any signal for Ca_V_1.3α_1_ in the Western blot analysis, suggesting that the kinase does not interact directly with the ion-conducting subunit of the channel complex. In contrast, the Western blot signal for Ca_V_β_3_ revealed a band above the 50 kDa size marker (expected size ~60 kDa), confirming the interaction between the kinase and the channel ancillary subunits.

Next, we aimed to investigate if the overexpression of the two LRRK2 variants could influence the expression of the channel’s subunits, particularly Ca_V_β_3_. Our Western blot analysis showed that neither LRRK2^WT^ nor LRRK2^G2019S^ significantly affected the expression of the channel’s main or auxiliary subunits (Figure 2b). Consequently, changes in Ca_V_β_3_ expression can be ruled out as a variable that might explain the results of the functional assays discussed below.

### 2.4. The Mutant LRRK2 Regulates L-Type Ca_V_1.3 Channel Functional Expression

The possible functional regulation of LRRK2 on Ca_V_1.3 channels was investigated through whole-cell patch-clamp recordings obtained in HEK-293 cells expressing the Ca_V_1.3α_1_, Ca_V_β_3_, and Ca_V_α_2_δ-1 subunits in the presence or absence of LRRK2^WT^ or its mutant LRRK2^G2019S^. It is important to highlight that this mutation is the most commonly identified genetic determinant associated with PD. Numerous structure–function studies support the notion that this variant is a gain-of-function mutant. This suggests a potential gain-of-toxic-function mechanism as a molecular pathogenic contributor to the disease [17,18,19,20]. Our investigation used HEK-293 cells as the expression system in our experiments because this cell line lacks endogenous Ca_V_ channels [21].

**Figure 3:**
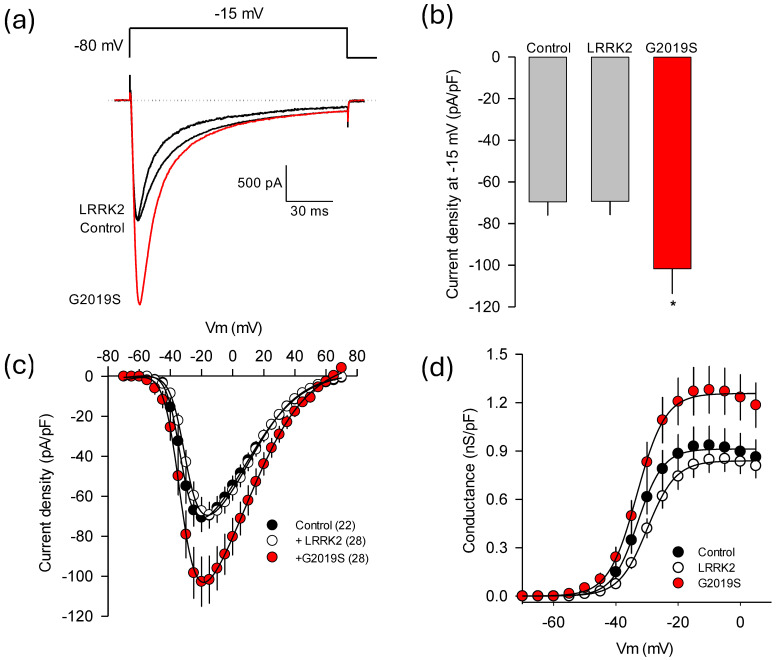
Regulation of the functional expression of L-type channels (Ca_V_1.3) by LRRK2^G2019S^ (**a**) Typical whole-cell patch-clamp *I*_Ca_ recordings obtained in HEK-293 cells expressing the Ca_V_1.3α_1_/Ca_V_β_3_/Ca_V_α_2_δ-1 channel subunits in the presence of both kinase variants (WT and G2019S). The currents were generated by depolarizing pulses to −15 mV from a *V*_h_ of −80 mV. (**b**) Average current density measured in HEK-293 cells expressing the Ca_V_1.3 channels in the presence of the LRRK2 variants as indicated. The asterisk denotes a significant difference (*p* < 0.05). (**c**) Average *I*-*V* curves for *I*_Ca_ obtained from cells expressing the Ca_V_1.3α_1_, Ca_V_β_3_, and Ca_V_α_2_δ-1 channels with LRRK2^WT^ and LRRK2^G2019S^ (*n* = 22–28 cells). The currents were generated by applying 5 mV activating steps from a *V*_h_ of −80 mV to potentials from −70 to +70 mV. (**d**) The voltage dependence of channel activation was assessed in control cells and cells co-transfected with both variants of the LRRK2 kinase. Conductance values were obtained from (**c**), as detailed in the Methods section, and the fitting parameters of the curves were similar across the three experimental conditions: *V*_1/2_ = −31.9, −29.9, and −31.9; *k* = 4.2, 4.9, and 4.7 for the control condition, LRRK2^WT^, and LRRK2^G2019S^, respectively.

Figure 3a shows representative whole-cell inward Ca^2+^ current (*I*_Ca_) traces recorded in the presence of LRRK2^WT^, its mutant LRRK2^G2019S^, or under control conditions. Transected HEK-293 cells generated robust macroscopic *I*_Ca_ through recombinant Ca_V_1.3 channels. Notably, overexpression of the LRRK2^G2019S^ variant significantly increased current amplitude, while LRRK2^WT^ did not produce an effect. In Figure 3b, the maximum current density (pA/pF) through the recombinant Ca_V_1.3 channels obtained by applying depolarizing pulses at −15 mV is compared. The result of this analysis in 22–28 cells confirmed that LRRK2^WT^ does not exert a noticeable effect on the magnitude of the currents. In contrast, overexpression of LRRK2^G2019S^ led to a significant increase of ~30% in maximum current density.

Figure 3c illustrates the average current density-voltage (*I*-*V*) curves, represented as the maximum current amplitude normalized to membrane capacitance (*C*_m_), in response to 140 ms depolarizing pulses from a *V*_h_ of −80 mV. The voltage was stepped in 5 mV intervals from −70 to +70 mV. Again, no significant differences in *I*_Ca_ densities were observed under control conditions (in the absence of LRRK2) or in the presence of LRRK2^WT^. In contrast, the *I*_Ca_ density through L-type Ca_V_1.3 channels in HEK-293 cells overexpressing the LRRK2^G2019S^ variant exhibited the same voltage dependence as those recorded with LRRK2^WT^; however, these currents were significantly larger at most of the voltages tested. To achieve a more comprehensive understanding of the abnormal regulation induced by LRRK2^G2019S^ on channel functional activity, we compared the gating properties of the Ca_V_1.3 channels heterologously expressed in HEK-293 cells under control conditions with those in cells overexpressing the two variants of the kinase. Thus, the voltage dependence of activation was estimated from the *I*-*V* curves shown in panel 3c by calculating conductance, as detailed in the Methods section. Conductance values were plotted against membrane potential in Figure 3d and fitted using Boltzmann equations.

### 2.5. Relevance of the Ca_V_β_3_ Subunit in the Effects of LRRK2^G2019S^

**Figure 4:**
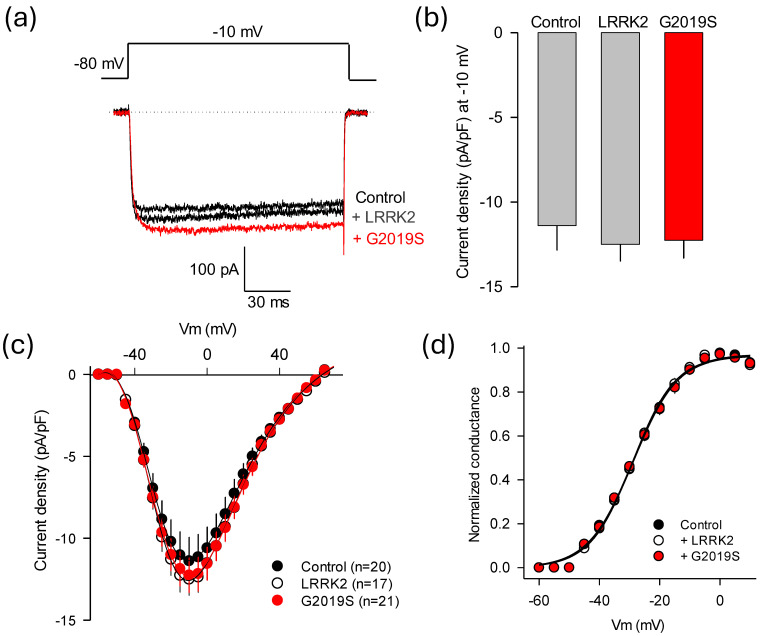
The regulatory effect of LRRK2^G2019S^ on the channels is absent without the auxiliary subunit Ca_V_β_3_. (**a**) Typical traces of *I*_Ba_ obtained from HEK-293 cells transfected with cDNAs encoding L-type channels Ca_V_1.3 (Ca_V_1.3α_1_/Ca_V_α_2_δ-1) without the auxiliary subunit Ca_V_β_3_. The currents were evoked by activating pulses to −10 mV from a *V*_h_ of −80 mV. (**b**) Average I_Ba_ density measured in control cells and in the presence of the LRRK variants, as in panel (**a**). (**c**) Average maximum currents graphed against the test potential in the experimental conditions, as indicated. The currents were evoked by applying voltage-activating pulses ranging from −70 to +70 mV, starting from a *V*_h_ of −80 mV. The number of recorded cells is indicated in parentheses. (**d**) Voltage dependence of activation for L-type channels (Ca_V_1.3α_1_/Ca_V_α_2_δ-1) in control cells and those expressing different variants of LRRK2. Conductance data were derived from panel (**c**), and the fitting parameters of the curves were similar across the three experimental conditions: *G*_max_ = 0.97, 0.96, and 0.96; *V*_1/2_ = −28.7, −29, and −29; *k* = 7.4, 7.0, and 7.4 for the control condition, LRRK2^WT^, and LRRK2^G2019S^, respectively. The error bars are hidden within the symbols.

Co-immunoprecipitation assays in Figure 2a confirm earlier findings that LRRK2 interacts with the auxiliary subunit Ca_V_β_3_ [9]. This suggests that Ca_V_β can directly modulate channel function and can also be a component of a regulatory protein complex that includes the LRRK2 kinase. Therefore, without the Ca_V_β subunit, this interaction could be disrupted, affecting channel regulation by the mutant kinase. To further investigate this, we examined whether the absence of Ca_V_β_3_ would affect the ability of LRRK2^G2019S^ to modulate current through Ca_V_1.3 channels heterologously expressed in HEK-293 cells. Figure 4a displays typical traces of Ba^2+^ currents (*I*_Ba_) through recombinant channels including only the ion-conducting subunit and the auxiliary Ca_V_α_2_δ-1 subunit (excluding Ca_V_β_3_) in the presence of either LRRK2^WT^ or the LRRK2^G2019S^ variant. In this series of experiments, Ba^2+^ replaced Ca^2+^ because the currents were small without Ca_V_β_3_, and it is well known that Ba^2+^ has higher permeability and conductance through Ca^2+^ channels than Ca^2+^ ions [22]. The results of this analysis indicate that the kinase regulatory effect is abrogated in the absence of Ca_V_β_3_, suggesting that this protein is essential for LRRK2^G2019S^ to modulate channel activity. Consistent with this, Figure 4b shows that overexpression of either LRRK2 variant does not result in substantial changes in current density in response to activating pulses to −10 mV from a *V*_h_ of −80 mV through Ca_V_1.3 channels lacking the Ca_V_β_3_ subunit.

Figure 4c shows the average voltage–current density relationships in response to 140 ms activating pulses from a *V*_h_ of −80 mV. In this series of experiments, like those presented in Figure 3c, the voltage increased in 5 mV steps from −70 to +70 mV. No significant differences in *I*_Ba_ densities were observed across any of the experimental conditions, reinforcing the crucial role of the Ca_V_β_3_ subunit in channel activity regulation by the mutant kinase. Next, we examined the gating properties of the channels more closely to see whether the abnormal regulation by LRRK2^G2019S^ affects the voltage dependence and kinetics of the currents. We analyzed the activation properties of Ca_V_1.3 channels expressed in HEK-293 cells under control conditions and in cells overexpressing the two variants of LRRK2. The results show no significant statistical differences in maximum conductance (Figure 4d).

### 2.6. Identification of Molecular Elements Implicated in the Regulation of Ca_V_1.3 Channels by LRRK2

**Figure 5:**
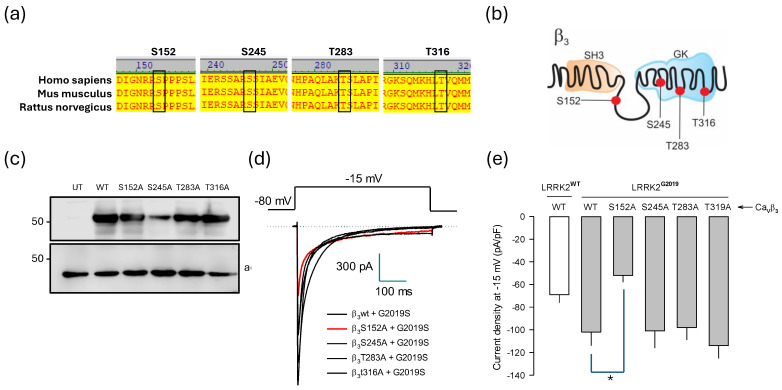
Molecular determinants of the Ca_V_1.3 channel regulation by LRRK2^G2019S^. (**a**) Amino acid sequence alignment of selected regions within the Ca_V_β_3_ subunit, illustrating the putative LRRK2 phosphorylation sites that are conserved across three species as indicated. The red black boxes indicate the sequence corresponding to the consensus phosphorylation site for the kinase. Automated sequencing of the PCR products verified that the constructs produced through site-directed mutagenesis of the LRRK2 phosphorylation site incorporated the intended mutant. (**b**) Localization of serine residues potentially phosphorylatable by LRRK2 across the different domains of the Ca_V_β_3_ protein sequence. (**c**) Western blot of protein extracts from HEK-293 cells co-expressing the wild-type or the mutant variants of Ca_V_β_3_ (S152A, S245A, T283A, and T316A) showing robust expression of all mutations in the heterologous system. (**d**) Representative Ca^2+^ currents through recombinant Ca_V_1.3α_1_/Ca_V_β_3_/Ca_V_α_2_δ-1 channels recorded in HEK-293 cells co-expressing the wild-type Ca_V_β3 or its phosphorylation variants in the presence of LRRK2^WT^ or its variant LRRK2^G2019S^. The currents were generated by 500 ms pulses at −15 mV, starting from a *V*_h_ of −80 mV. (**e**) Average *I*_Ca_ density in HEK-293 cells expressing Ca_V_1.3α_1_/α_2_δ-1 channels along with the wild-type Ca_V_β_3_ subunit or its phosphorylation mutants (filled bars). *I*_Ca_ density was determined from the current amplitude after the application of a depolarizing pulse of −15 mV from a *V*_h_ of −80 mV, normalized to *C*_m_, in the presence of LRRK2 or LRRK2^G20219S^, as indicated (*n* = 13–20 cells). The open bar shows the average *I*_Ca_ amplitude through recombinant Ca_V_1.3 channels containing the wild-type Ca_V_β_3_ subunit and in the presence of LRRK2 WT, and it is identical to what is presented in Figure 3b for the purposes of comparison. The asterisk indicates a statistically significant difference (*p* < 0.05).

To determine the molecular elements underlying the regulation of Ca_V_1.3 channels by LRRK2^G2019S^, we first examined residues in the Ca_V_β_3_ subunit that the kinase could potentially phosphorylate (Figure 5a). Bioinformatics analysis identified four possible phosphorylation sites: S152, S245, T283, and T316. Through multiple sequence alignments, we confirmed that all these sites are conserved across various species, including humans (Figure 5a). Notably, three of the potential phosphorylation sites are in the GK region, while S152 is located in the HOOK domain of the Ca_V_β_3_ subunit (Figure 5b). To investigate the role of these phosphorylatable residues in regulating channel activity by LRRK2^G2019S^, we expressed the wild-type Ca_V_β_3_ auxiliary subunit alongside its mutated variants in HEK-293 cells and conducted an initial analysis on them using Western blotting. The results demonstrated that this heterologous system expressed the wild-type protein and the generated mutants robustly (Figure 5c).

Next, we examined the functional role of the four mutant constructs where serine or threonine residues were replaced with alanine. The mutants were transiently co-expressed with the ion-conducting Ca_V_1.3α_1_ protein and the Ca_V_α_2_δ-1 ancillary subunit in HEK-293 cells. Whole-cell patch-clamp recordings were used to assess how these mutations affected the channel’s functional expression (Figure 5d). The results showed that three of the four mutant constructs exhibited no significant changes in the regulation of the channels mediated by LRRK2^G2019S^. In contrast, the S152A mutant prevented the up-regulation caused by the mutant kinase when co-expressed with the Ca_V_1.3α_1_/Ca_V_α_2_δ-1 channels. Figure 5e compares the maximum current density values obtained at −15 mV from at least n cells and confirms that S152 is a phosphorylation target of the mutant kinase. Consistent with this, replacing S152 with a non-phosphorylatable amino acid completely abolishes the actions of LRKK2^G2019S^ on Ca_V_1.3 channels.

**Figure 6:**
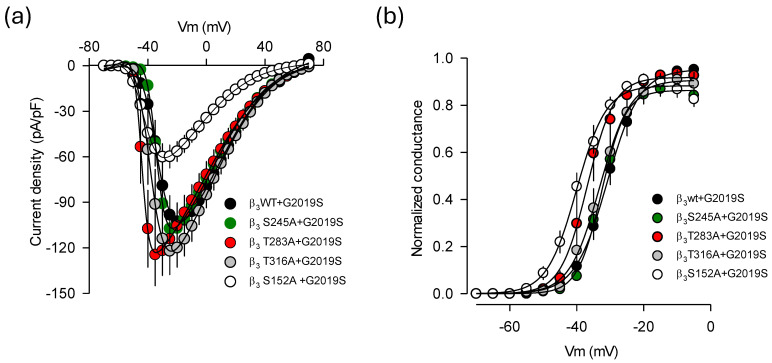
Effects of the phosphorylation mutants of the Ca_V_β_3_ subunit on current voltage dependence and density. (**a**) Superimposed average ± SEM *I*-*V* curves for *I*_Ca_ obtained from HEK-293 cells expressing Ca_V_1.3 channels comprising the wild-type auxiliary Ca_V_β_3_ subunit and its phosphorylation mutants (S152A, S245A, T283A, and T316A) in the presence of LRRK2^G2019S^, as indicated. *I*_Ca_ density was estimated from a series of activating pulses between −70 and +70 mV from a *V*_h_ of −80 mV, in 5 mV steps (*n* = 14–20 cells). (**b**) The amplitude of *I*_Ca_ was then normalized to their corresponding *C*_m_ values, and activation curves were constructed with the data derived, shown in panel (**a**). The fitting parameters of the curves are as follows: *G*_max_ = 0.95, 0.88, 0.86, 0.92, and 0.91; *V*_1/2_ = −30.9, −40.1, −32.6, −36.8, and −33.1; k = 4.8, 4.4, 3.5, 4.0, and 4.5 for Ca_V_β_3_^WT^, Ca_V_β_3_^S152A^, Ca_V_β_3_^S245A^, Ca_V_β_3_^T283A^, Ca_V_β_3_^T319A^, respectively.

The analysis of the voltage dependence of the currents through the Ca_V_1.3 channel containing the phosphorylation mutations in the Ca_V_β_3_ sequence, including S152A, revealed a shift in the hyperbolizing direction of the *I-V* curves of about 10 mV in cells co-expressing LRRK2^G2019S^ compared to those expressing the wild-type Ca_V_β_3_ (Figure 6a,b). This finding is not unexpected, as phosphorylation of specific residues in the Ca_V_β subunits can lead to functional changes in the Ca_V_α_1_ subunits. Of particular interest, site-directed mutagenesis of conserved phosphorylation residues has shown that the S152E variant, which mimics the phosphorylation of the Ser residue in Ca_V_β_2b_, affects the current density for cardiac Ca_V_1.2 (L-type) channels and shifts the activation and inactivation curves toward more depolarizing potentials. In contrast, the S152A mutation, which prevents phosphorylation, displayed opposing effects [23,24]. Furthermore, it has been found that LRRK2 causes a significant hyperpolarizing shift in the activation curve of Ca_V_2.1 channels, supporting the idea that LRRK2 directly modulates Ca_V_2.1 channels’ activity by increasing the proportion of channels available to be opened at more hyperpolarized membrane potentials [9]. Our data align with these findings. Notably, the hyperpolarizing shift in the voltage dependence of currents may explain the slight decrease in current density recorded at −15 mV in the presence of LRRK2^G2019S^ compared to that observed with LRRK2^WT^ co-expression (see Figure 5e). However, this change in channel activation alone cannot fully account for the effects of the S152A mutation in response to LRRK2^G2019S^. This strongly suggests that the absence of phosphorylation is primarily responsible for most of the effects of this mutation in Ca_V_β_3_.

**Figure 7:**
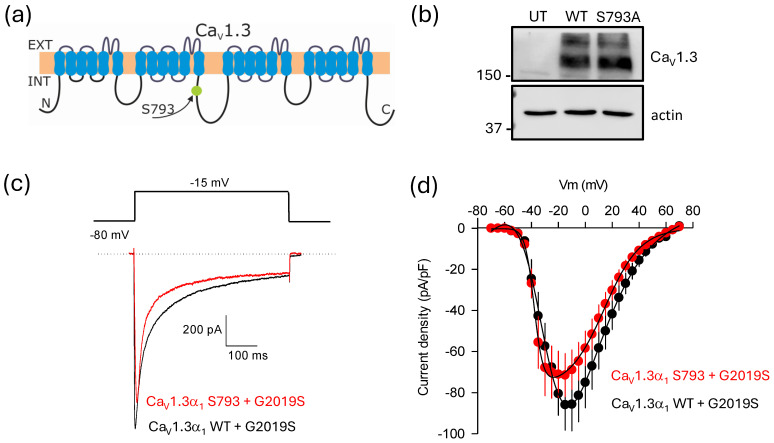
The regulatory effect of LRRK2^G2019S^ on Ca_V_1.3 channels may not involve the main Ca_V_1.3α_1_ subunit. (**a**) The schematic representation of the Ca_V_1.3α_1_ ion-conducting subunit highlights a serine residue at position 793, which LRRK2 may phosphorylate. This residue is situated in the intracellular loop connecting the repeated domains II and III of the channels. (**b**) Western blot analysis was conducted on proteins from untransfected HEK-293 cells (UT; lane 1) and cells expressing Ca_V_1.3α_1_/Ca_V_β_3_/Ca_V_α_2_δ-1 channels (lanes 2 and 3), using an antibody that recognizes the Ca_V_1.3α_1_ protein. The blot reveals two bands above the 150 kDa marker; one of these bands, likely the upper one, has not been observed previously and may indicate a nonspecific interaction with the antibody. This band is present in both lanes corresponding to the WT protein and the S793A mutant, suggesting that it might not impact te interpretation of the data. (**c**) Typical whole-cell currents obtained in HEK-293 cells co-expressing the Ca_V_1.3α_1_/Ca_V_β_3_/Ca_V_α_2_δ-1 channel subunits in the presence of both kinase variants (WT and G2019S). The currents were elicited by depolarizing pulses from a *V*_h_ of −80 to −15 mV, as indicated. (**d**) Average *I*-*V* curves for *I*_Ca_ were obtained from cells expressing Ca_V_1.3 channels in conjunction with the two variants of the LRRK2 kinase (*n* = 16–23 cells). The currents were elicited by applying 5 mV activating steps from a *V*_h_ of −80 mV to potentials from −70 to +70 mV.

On the other hand, though the pore-forming subunit Ca_V_1.3α_1_ did not show a physical interaction with either LRRK2 variant in our co-immunoprecipitation assays, bioinformatics analysis identified a serine residue at position 793 as a potential phosphorylation site for LRRK2 (Figure 7a). To investigate whether the kinase could phosphorylate this site, we created a mutation that substituted the serine at position 793 with alanine using site-directed mutagenesis. We then assessed the effects of this mutation through electrophysiological recordings. The presence of the amino acid substitution was confirmed by automated sequencing, and its protein expression was validated by Western blot of lysates from transfected HEK-293 cells (Figure 7b).

The current traces presented in Figure 7c were obtained from cells cotransfected with a construct encoding wild-type Ca_V_1.3α_1_ cDNA, along with the auxiliary subunits (Ca_V_β_3_/Ca_V_α_2_δ-1). Another batch of HEK-293 cells was transfected with the mutant construct (S793A) and the identical auxiliary subunits. Notably, the recombinant channels harboring the mutation showed robust expression levels and generated the typical current waveforms characteristic of wild-type Ca_V_1.3 channels. Additionally, co-expression of the LRRK2^G2019S^ mutant did not significantly affect the current amplitudes in either condition, suggesting that the mutant channels maintained kinase regulation and that S793 in Ca_V_1.3α_1_ may not be directly involved in this regulatory process. Although there was a minor effect in the voltage dependence of channel activation (~5 mV) and a tendency for the current density through the mutant channels to decrease by about 16%, these changes were not statistically significant. While additional research is necessary to determine whether S793 is playing a role in the regulation of the channels by LRRK2^G2019S^, as discussed below, the findings from the study of the Ca_V_β auxiliary subunit mutants could exclude a possible contribution of S793 in the pore-forming Ca_V_1.3α_1_ to this regulation. In line with this, the voltage–current density relationships depicted in Figure 7d showed no significant statistical differences in current densities between cells expressing the wild-type and the mutant Ca_V_1.3α_1_ pore-forming subunit in the presence of LRRK2^G2019S^.

## 3. Discussion

Protein phosphorylation is a post-translational mechanism that regulates biomolecules. It is a discrete reversible change but can potentiate cellular activity. Protein kinases catalyze the addition of phosphate moieties to serine, tyrosine, or threonine residues since they have a nucleophilic group that can attack the universal donor phosphate, adenosine triphosphate (ATP). Ca_V_ channels, as one of the most relevant regulators of calcium entry into cells and, therefore, of Ca^2+^-dependent signaling, are a common target of kinases. The actions of various kinases on Ca_V_ channels, such as PKA, PKC, PKG, and Cdk5, among others, are currently documented [25,26,27,28,29].

Within this context, it was previously described that channels of the Ca_V_2.1 class could be the target of the LRRK2 kinase and that the phosphorylation of the Ca_V_β_3_ auxiliary subunit regulates the release of neurotransmitters. Since Ca_V_β_3_ can be part of other Ca_V_ channels [30], we sought to determine whether this subunit, as a component of Ca_V_1.3, could confer regulation by LRRK2. The Ca_V_1.3 channels are expressed in the dopaminergic neurons of the SNc, contributing to the excitation–transcription process. Furthermore, by having a lower activation threshold than other HVA channels [31,32,33], Ca_V_1.3 channels cause the neurons in which they are expressed to function as pacemaker cells and can spontaneously generate action potentials [4,8,34].

Our study revealed that the L-type channels of the Ca_V_1.3 class can be regulated by LRRK2 through the phosphorylation of the Ca_V_β_3_ subunit, in an analogous manner as previously reported for presynaptic Ca_V_2.1 channels [9]. However, for Ca_V_1.3 channels, this regulation occurs only when the mutant form LRRK2^G2019S^ is expressed. Under these conditions, phosphorylated Ca_V_1.3 channel complexes may be delivered more efficiently to the membrane or stabilized on the cell surface through protein–protein interactions, thereby enhancing the Ca^2+^ current [26,35,36,37]. Additionally, alterations in phosphorylation may lead to changes in the interactions between Ca_V_β and proteins involved in the degradation pathways, which could influence the lifespan of channels at the cell membrane [38,39]. These findings could be relevant for unveiling novel aspects of dopaminergic neurons’ function and the role of the mutant kinase LRRK2^G2019^ in PD.

The interaction of LRRK2^WT^ kinase with the Ca_V_β_3_ subunit of Ca_V_2.1 channels increases current through presynaptic channels, suggesting that the kinase can influence their function through phosphorylation. However, our findings indicate that this regulation may be specific to the Ca_V_2.1 channel and not extend to other channel subtypes, such as Ca_V_1.3, under physiological conditions. It may only occur when the kinase is more active or has a gain-of-function mutation. The reason for this difference is unclear but could be associated with the distinct microenvironment of the channels, which might have different molecular interactors or participate in separate signaling pathways. Here, it is worth noting that, in contrast to Ca_V_2.1 presynaptic channels that are involved in neurotransmission, Ca_V_1.3 channels contribute to determining the excitability of dopaminergic neurons by controlling their pacemaker activity. Therefore, regulating Ca_V_2.1 channels by LRRK2^WT^ may involve unique domains that enable specific interactions with proteins linked to the presynaptic channel complex, which are not present in Ca_V_1.3 channels with a different function and subcellular location. This also may help clarify why LRRK1, which, despite being homologous to LRRK2, seemingly lacks the internal domains essential for effective interaction with Ca_V_ channels [9]. In summary, the ability of LRRK2^WT^ to phosphorylate and regulate Ca_V_2.1 channels, while having minimal impact on other subtypes such as Ca_V_1.3, may arise from specific protein interactions, unique structural domains within LRRK2, and the distinct physiological roles of these channels in neuronal signaling.

On the other hand, it is widely accepted that Ca_V_1.3 channels participate in PD [8,40,41,42,43]. The main characteristic of PD is the progressive loss of dopaminergic neurons, primarily in the SNc. While the cause of this selective vulnerability in this cell population is presently unclear, the blockade of L-type channels (Ca_V_1) with dihydropyridines (DHPs) seems to protect dopaminergic neurons against the neurotoxins rotenone and MPTP [43]. Furthermore, the protective effect of DHPs is also present in animal models of PD caused by 6-OH dopamine and MPTP administration [43,44,45]. However, evidence for the specific contribution of Ca_V_1.3 channels to PD comes from brain slices of knockout Ca_V_1,3 mice, which exhibited significantly less dendritic fragmentation induced by rotenone compared to wild-type animals [34]. While direct evidence for the role of Ca_V_1.3 channels in neuronal degeneration is still lacking, previous studies have proposed that these channels are determinants for autonomous pacing in dopaminergic neurons [33,35,46]. Therefore, the contribution of the Ca_V_1.3 channels to the peacemaking activity has been proposed as a specific factor for neuronal susceptibility to neurodegeneration.

Therefore, targeting the L-type channels may represent a promising therapeutic approach for Parkinson’s disease (PD). The dihydropyridine (DHP) antagonist, isradipine, seemingly slows the progression of the motor symptomatology in patients with PD. While this represents a significant advancement in treatment options, recent findings have raised questions regarding this approach’s efficacy and long-term benefits [47]. Likewise, since Ca_V_1.3 channels are largely expressed in the SNC, where they have been associated with increased neuronal vulnerability, selective blockers for these channels could optimize neuroprotective effects while reducing the cardiovascular side effects of DHPs. This distinction is crucial because neuronal Ca_V_1.3 channels are pharmacologically different from cardiac Ca_V_1.2 channels. Notably, the IC_50_ for blockade of Ca_V_1.3 by nimodipine is 20 times higher than that for Ca_V_1.2 [48]. Additionally, given the complexity of PD and Ca^2+^ signaling in dopaminergic neurons of the SNc, combining Ca_V_1.3 channel inhibitors with other neuroprotective drugs may also be beneficial [40].

**Figure 8:**
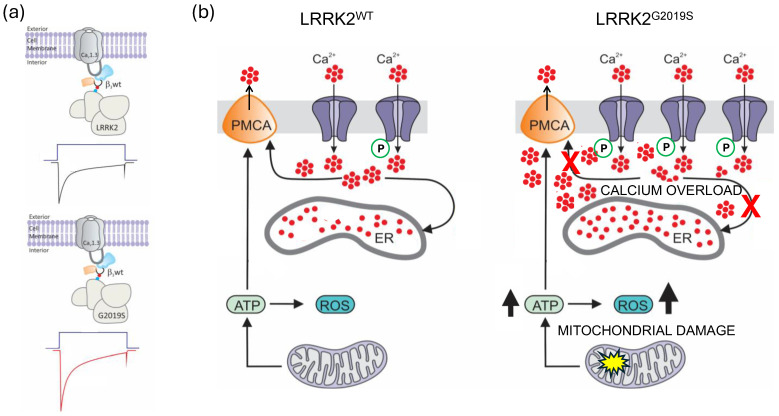
The mutant protein LRRK2^G2019S^ can alter the function of Ca_V_1.3 channels because the auxiliary subunit Ca_V_β_3_ is essential for their expression and regulation. (**a**) Phosphorylation of Ca_V_β_3_ enhances the intracellular transport of the channel complex to the cell membrane, stabilizes the channel complex, and prevents degradation. This modification is crucial for proper calcium signaling in neurons. The G2019S mutation in LRRK2 increases its activity approximately twofold compared to the wild-type kinase, which is key to its pathogenic effects. The hyperactivity of this protein may lead to an increase in the number of Ca_V_1.3 channels at the neuronal membrane. (**b**) Since Ca_V_1.3 channels control the rhythmic firing of dopaminergic neurons in the substantia nigra, the increased expression at the membrane could affect pacemaker frequency and elevate intracellular calcium levels. Even more, the hypothesis that the progression of PD could be associated with a dysregulation of Ca_V_1.3 channels due to increased LRRK2 activity is consistent with the idea that, during the development of the disease, there is an increase in the intracellular calcium concentration, which demands extra effort from the calcium reuptake and extrusion mechanisms. Likewise, it is also recognized that, in PD, the functionality of certain calcium buffering proteins is disrupted. This, in conjunction with an increase in energy demand and oxidative stress, results in mitochondrial damage and ultimately in the loss of dopaminergic cells in the SNc.

Last, as mentioned above, the LRRK2^G2019S^ mutation represents one of the most common genetic causes associated with PD. Specifically, this mutation is found in nearly 2% of patients with apparently sporadic PD, and its prevalence doubles in patients with a family history of the disease [19]. This makes it one of the most significant mutations in the genetic context of the disease. As our work suggests, the mutated form of LRRK2^G2019S^ increases the kinase’s ability to phosphorylate Ca_V_1.3 channel proteins, which can disrupt cell function and may contribute to neuronal degeneration. Figure 8 provides a hypothesis of the effects of alterations in Ca_V_1.3 function due to the gain-of-toxic function of LRRK2^G2019S^ kinase in PD. Dysregulation of the channels can disrupt intracellular Ca^2+^ homeostasis, probably by altering their role in the pacemaker activity of dopaminergic neurons. When Ca^2+^ clearance systems become overwhelmed, the previously mentioned changes can result in significant alterations in cellular metabolism and elevated production of reactive oxygen species. These processes can damage mitochondria and promote α-synuclein aggregation.

## 4. Materials and Methods

### 4.1. Bioinformatics

The in silico analysis of the potential phosphorylation sites by LRRK2 in the sequences encoding the Ca_V_1.3α_1_ and Ca_V_β_3_ subunits was performed using freely accessible databases and bioinformatic tools on the Internet (GPS 6.0—Kinase-specific Phosphorylation Site Prediction software v.6.0 available at the URL https://gps.biocuckoo.cn/). Once the phosphorylation sites were identified, we replaced them with non-phosphorylatable residues (alanines).

### 4.2. Cell Culture and Transfection

HEK-293 cells (ATCC; Cat. # CRL-1573) were seeded in 35 or 100 mm Petri dishes using a DMEM culture medium complemented with 5% fetal bovine serum, 2 mM L-glutamine, 110 mg/L of Na^+^ pyruvate, 100 µg of streptomycin, and 100 U/mL of penicillin. Cell cultures were maintained at 37 °C in an air atmosphere with 5% CO_2_ and 80% relative humidity. All cDNA clones used in this work were analyzed using endonucleases to determine their identity and integrity as stipulated in the corresponding restriction maps and by automatic sequencing using the BigDye Terminator v3.1 Cycle Sequencing Kit (Applied Biossystems, Waltham, MA, USA). The Ca_V_1.3α_1_ clone was kindly provided by Dr. Diane Lipscombe (Brown University, Providence, RI, USA); the Ca_V_β_3_ and Ca_V_α_2_δ-1 clones were a generous gift of Dr. Kevin P. Campbell (The University of Iowa, Iowa City, IA, USA). The wild-type LRRK2 (LRRK2^WT^) cDNA clone and its LRRK2^G2019S^ mutant were purchased from Addgene (Watertown, MA, USA, catalogs #25044 and 25045, respectively). For the Western blot and immunoprecipitation assays, cells were transfected using Lipofectamine 2000 (Invitrogen, Waltham, MA, USA). For the electrophysiology experiments, a plasmid coding for the green fluorescent protein (GFP; pGeen Lantern-1) was also included in the transfection reaction to recognize the transfected cells. Cells were dispersed 24 h before transfection and plated in 35 mm Petri dishes to reach ~60% confluency. Transfections were carried out using the TurboFect reagent (Thermo Fischer Scientific, Waltham, MA, USA) with 1.2 μg of the cDNA clones of Ca_V_1.3α_1_, Ca_V_β_3_, Ca_V_α_2_δ-1, and LRRK2^WT^ kinase or its LRRK2^G2019S^ mutant, according to the manufacturer’s instructions. At 24 h, a medium change was performed with a fresh supplemented medium, and, at 2 d after transfection, cells were prepared for use.

### 4.3. Immunofluorescence

To determine the expression and distribution of the two variants of the LRRK2 kinase, the HEK-293 cells were seeded onto poly-L-lysine pretreated coverslips. After 24 h, the cells were transiently transfected with the LRRK2^WT^-GFP and LRRK2^G2019S^-GFP constructs. Following an additional 48 h incubation, the cells were washed, fixed with 4% paraformaldehyde, and permeabilized with PBS plus 0.2% Triton X-100. The coverslips were then mounted using VECTASHIELD with DAPI (H-1200) and analyzed for fluorescence using confocal microscopy (Leica TCS SP8, Wetzlar, Germany).

### 4.4. Site-Directed Mutagenesis

The serine or threonine residues identified in the Ca_V_1.3α_1_ and Ca_V_β_3_ sequences as possible targets of the LRRK2 phosphorylation were replaced by non-phosphorylatable residues. Mutagenic oligonucleotides were designed to introduce point mutations into the sequence of both subunits of the Ca_V_1.3 channels (Ca_V_α_1_ and Ca_V_β_3_) using the commercial QuikChange XL Site-Directed Mutagenesis (QIAGEN, Hilden, Germany) kit following the manufacturer’s instructions. The sequence of oligonucleotides is given in Table S1. Briefly, the reaction utilized 150 ng of template DNA, 125 ng of primers, and 1 µL of HF DNA polymerase (2.5 U/μL). The reaction of amplification involved the following steps: one cycle of denaturation at 95 °C for 1 min; 18 cycles of 95 °C for 50 s, 60 °C for 50 s, and 68 °C for 8 min, followed by one cycle at 68 °C for 7 min. After amplification, to digest the parental DNA, 1 μL of the enzyme Dpn I (10 U/µL) was added, and the reaction was incubated at 37 °C for 1 h. Subsequently, DH5α cells were transformed, subjected to alkaline lysis for plasmid extraction, and analyzed through automated sequencing.

### 4.5. Western Blotting

A RIPA buffer was used to prepare the lysates of HEK-293 cells. Protein concentration was determined by means of the bicinchoninic acid method. Samples were then denatured using a loading buffer with SDS plus β-mercaptoethanol and incubated at 95 °C for 5 min. The proteins were then separated by electrophoresis on 8–10% polyacrylamide gels and subsequently transferred to nitrocellulose membranes. Subsequently, membranes were incubated with non-fat dry milk (NDFM) or 3–5% BSA in TBS-Tween for 1–2 h before being incubated with the primary antibody diluted in TBS-Tween supplemented with NDFM or 3–5% BSA. The antibodies used in these assays are shown in Supplementary Table S2. After washing, nitrocellulose membranes were blocked with NDFM-TBS-Tween and incubated with secondary antibodies. Chemiluminescence was revealed using the Odyssey system (LI-COR Biosciences, Lincoln, NE, USA).

### 4.6. Immunoprecipitation

As mentioned earlier, HEK-293 cells were transiently transfected with the cDNA plasmids of interest, and protein extracts were prepared. Once the cell lysates were obtained, 500 μL of lysis buffer (50 mM Tris-HCl pH 8.0; 150 mM NaCl; 1% Triton) was added. After centrifugation, the supernatants were discarded. Protein extracts were pre-cleared using 0.5–1.5 mg of total proteins for each assay in a mixture containing pre-equilibrated PGA and lysis buffer. Samples were centrifuged again, and the PGA pellets were discarded. A total of 2.5 μg of primary antibodies was used, and an unrelated antibody was employed for the negative control (Supplementary Table S1). The primary antibodies were incubated overnight at 4 °C with constant shaking. Once the washes were completed, the samples were centrifuged, and the supernatant was removed. Finally, the pellet was incubated at 95 °C for 5 min in 20 μL of loading buffer. After denaturation, the samples were centrifuged, followed by the execution of the Western blot analysis.

### 4.7. Electrophysiology

HEK-293 cells were transiently transfected with Ca_V_1.3 channels along with LRRK2 (and their mutants) and then subjected to electrophysiological assays employing the whole-cell patch-clamp technique [49]. Coverslips with the cells were positioned in the electrophysiological recording chamber, which contained 500 μL of the bath solution, composed of (in mM): 130 TEACl, 5 CaCl_2_ (or 10 BaCl_2_, as indicated), 10 HEPES, and 10 glucose (pH 7.3 and osmolarity of ~300 mOsm). The pipette was filled with a solution consisting of (in mM) 125 CsCl, 5 MgCl_2_, 10 HEPES, 10 EGTA, 0.1 GTP, and 4 ATP (pH 7.3 and osmolarity of ∼290 mOsm). Data were collected using an Axopatch 200B amplifier (Molecular Devices, San Jose, CA, USA) connected to a Digidata 1440 interface (Molecular Devices) controlled by a personal computer. Pulse protocols, data acquisition, and analysis were conducted using pCLAMP (v10.7; Molecular Devices) and Sigma Plot (v12.5; SPSS) software. Ca_V_1.3 channel currents were evoked by applying depolarizing pulses of 140 ms duration from a holding potential (*V*_h_) of −80 mV. Residual leakage currents and capacitance were subtracted using a standard P/4 protocol. Current signals were filtered and digitized at 2 and 5 kHz, respectively. The voltage dependence of the currents was examined using a protocol that involved a series of 140 ms depolarizing test pulses ranging from −70 to +70 mV, in 5 mV increments. The maximum current was calculated for each voltage, and current–voltage relationships (*I*-*V*) were created.

### 4.8. Statistical Analysis

Data were analyzed and graphed by means of the Sigma Plot software (v12.5; SPSS) and are expressed as mean ± S.E.M., where *n* represents the number of recorded cells or assays performed. The statistical significance of the data was assessed using Student’s t-test to compare the means of two unpaired datasets. Differences were deemed statistically significant when the *p*-value was less than 0.05.

Current density was computed by dividing current amplitude (*I*) by cell capacitance (*C*_m_). Therefore, the current density measures the current flow per unit area of the cell membrane. The current values were converted to conductance using the following equation: *G* = *I*/(*V*_m_ − *V*_rev_), where *I* represents peak current, *G* denotes conductance, *V*_m_ is the test potential, and *V*_rev_ is the extrapolated reverse potential. Conductance–voltage (*G*-*V*) relationships for activation were fitted using a Boltzmann equation: *G* = *G*_max_/(1 + exp[(*V*_m_ − *V*_1/2_)/*k*] − 1), where *G*_max_ represents maximum conductance, *V*_m_ is the test potential, *V*_1/2_ is the voltage at which half-maximal activation of *G*_max_ occurs, and *k* is the slope factor.

## Data Availability

The data supporting the findings of this study can be obtained from the corresponding authors upon reasonable request.

## Supplementary Materials

The following supporting information is available for download at: https://www.mdpi.com/article/10.3390/ijms26073229/s1.
