# Supplementary material for: The Leucine-Rich Repeat Kinase 2 Variant LRRK2G2019S Up-Regulates L-Type (CaV1.3) Calcium Channel via the CaVβ3 Subunit: Possible Role in the Pathogenesis of Parkinson’s Disease
Source: Int J Mol Sci. 2025 Mar 31;26(7):3229. doi: 10.3390/ijms26073229 (PMC11989569; doi:10.3390/ijms26073229)
Supplement: Supplementary file 1 [file ijms-26-03229-s001.zip › ijms-3396768-supplementary.pdf]

Supplemental Table $S1$

S152A	Reverse	GGAGGAGGGGCACGTCGGTTGCCAATG
	Forward	CATTGGCAACCGACGTGCCCCTCCTCC
S245A	Reverse	GCTCTTCTGCCCGCGCCAGCATT
	Forward	CTCAGCAATGCTGGCGCGGGCAG
T283A	Reverse	ACAGCTAGCCAAGGCCTCACTGG
	Forward	GGGGCCAGTGAGGCCTTGGCTAG
T316A	Reverse	CCAGATGAAGCACCTCGCTGTACAGATGATGGC
	Forward	GCCATCATCTGTACAGCGAGGTGCTTCATCTGG

Supplemental Table S2

	Company	Catalogue number	Host	Clonality	Dilution	RRID
GFP	Novus	NB600-308	Rabbit	Polyclonal	1:5000	AB_2713963
LRRK2	abcam	ab133474	Rabbit	Monoclonal	1:5000	AB_10003058
$Ca_V\beta_3$	Alomone	ACC-008	Rabbit	Polyclonal	1:5000	AB_2039787
Ca_V1.3	Alomone	ACC-005	Rabbit	Polyclonal	1:1000	AB_2039775
actin	Genetex	GTX629630	Mouse	Monoclonal	1:10000	AB_2728646

Secondary antibodies

	Company	Catalogue number	Host	Clonality	Dilution	RRID
Anti-mouse	Jackson ImmunoResearch	115-035-003	Goat	Polyclonal	1:10000	AB_10015289
Anti-rabbit	Jackson ImmunoResearch	111-035-003	Goat	Polyclonal	1:10000	AB_2313567
